# How to Link Brain and Experience? Spatiotemporal Psychopathology of the Lived Body

**DOI:** 10.3389/fnhum.2016.00172

**Published:** 2016-04-28

**Authors:** Georg Northoff, Giovanni Stanghellini

**Affiliations:** ^1^University of Ottawa Institute of Mental Health ResearchOttawa, ON, Canada; ^2^Center for Cognition and Brain Disorders, Hangzhou Normal UniversityHangzhou, China; ^3^Center for Brain and Consciousness and Departments of Radiology and Psychiatry, Sheng Ho Hospital, Taipei Medical University (TMU)Taipei, Taiwan; ^4^College for Humanities and Medicine, Taipei Medical University (TMU)Taipei, Taiwan; ^5^Institute for Advanced Biomedical Technologies (ITAB), “G. d’Annunzio” University of Chieti-PescaraChieti, Italy; ^6^DiSPUTer, “G. d’Annunzio” University of Chieti-PescaraChieti, Italy; ^7^Department of Cognitive Science and Language, Diego Portales UniversitySantiago, Chile

**Keywords:** abnormal bodily phenomena, brain resting state, phenomenology, schizophrenia, spatio-temporal psychopathology

## Abstract

The focus of the present article is on sketching a psychopathology of the body in schizophrenia and linking it to brain activity. This is done providing converging data from psychopathological evidence (phenomenal), phenomenological contructs (trans-phenomenal) and neuroscientific measures (pre-phenomenal). The phenomenal level is the detailed documentation of the patients’ subjective anomalous experiences. These phenomena are explicit contents in the patients’ field of consciousness. The trans-phenomenal level targets the implicit yet operative matrix that underlies these anomalous subjective experiences. Abnormal phenomena are viewed as expressions of a modification of trans-phenomenal matrix, that is, in terms of an abnormal synthesis or integration through time of intero-, proprio- and extero-ceptive stimuli. Finally, we link the abnormalities of the trans-phenomenal matrix to pre-phenomenal alterations of the brain resting state and of its spatio-temporal organization, as documented by neurobiological methods providing spatial and temporal resolution of intrinsic brain activity (with many features of the resting state remaining yet unclear though). Based on phenomenological research, the body in schizophrenia is typically experienced in an itemized way as an object external to one’s self and unrelated to events in the external world. Based on neurobiological data, we tentatively hypothesize that such anomalies of the lived body are related to decreased integration between intero-, extero- and proprioceptive experiences by the brain’s spontaneous activity and its temporal structure. Taken all together, this suggests that we view abnormalities of bodily experience in terms of their underlying abnormal spatiotemporal features which, as we suppose, can be traced back to the spatiotemporal features of the brain’s spontaneous activity.

## Introduction

Psychopathological disorders are complex disturbances showing a wide variety of symptoms that cover most brain functions, including sensorimotor, affective, cognitive, and social functions. For instance, schizophrenic patients suffer from cognitive dysfunction (e.g., formal thought disorders), affective changes (e.g., inadequate or diminished affective modulation), social withdrawal (e.g., lack of attunement, inability of immersion in the world), and sensorimotor symptoms (e.g., catatonia).

Neuroimaging using techniques like functional magnetic resonance imaging (fMRI) and Electroencephalogram (EEG) have focused on extrinsic activity that concerns the brain’s response to sensorimotor, cognitive, affective or social stimuli or tasks, i.e., stimulus-induced or task-evoked activity. For all the progress in investigating the brain’s extrinsic activity and its various functions, diagnostic or therapeutic markers still remain elusive. In its search for these specific markers, recent neuroimaging in psychiatry has shifted to the brain’s *intrinsic* activity, its so-called resting state activity.

Roughly, the brain’s intrinsic or resting state activity describes the brain’s neural activity in the absence of any specific tasks or stimuli (Logothetis et al., 2009). However, the term “resting state” must be considered relative (rather than absolute) since even in the absence of specific stimuli or tasks, there is still plenty of processing going on. For instance, the interoceptive stimuli from the own body like the heart or the respiration continue to enter into the resting state (Duncan and Northoff, 2013; Weinberger and Radulescu, 2016); these are usually filtered out in subsequent resting state analyses but may nevertheless modify its ongoing dynamics (Duncan and Northoff, 2013; Northoff, 2014a). Moreover, there are plenty of cognitions in the form of task-unrelated thoughts or mind wandering, going on in the resting state (Smallwood and Schooler, 2015). Taken into account these (Weinberger and Radulescu, 2016, and others) different lines of processing, the resting state cannot really be conceived a proper rest in the literal form of the term. It may instead be rather conceived a state where the neural processing is directed more towards internal contents as related to the own body and cognitions rather than the external contents of the environment as when applying specific stimuli or tasks (Vanhaudenhuyse et al., 2011; Northoff, 2014a). When we speak of “resting state” in the following, we presuppose such more internally-directed state (as distinguished from a more externally-directed state) rather than a “true” resting state.

The brain’s resting state activity can spatially be characterized by various neural networks consisting of regions showing close “functional connectivity” yielding a particular spatial structure (see below for details; methodological issues like global signal regression in especially fMRI need to be considered though; Weinberger and Radulescu, 2016; Duncan and Northoff, 2013). The same applies to the temporal domain, where fluctuations in different frequency ranges are coupled with each other, providing “neural synchrony” (Engel et al., 2013). Neuroimaging reports a variety of changes in both functional connectivity and neural synchrony in various psychiatric disorders (that show low degrees of specificity and large heterogeneity though; see Weinberger and Radulescu, 2016). The exact meaning of these spatial and temporal abnormalities in the resting state, if real and based on alterations in physiological mechanisms, for psychiatric symptoms remains unclear though.

Based on the spatiotemporal features of the resting state and their alterations in psychiatric disorders, one of us (Northoff, 2015a,b) suggested a novel approach to psychopathology, called “*patiotemporal psychopathology*”. In a nutshell, such spatiotemporal psychopathology conceives psychopathological symptoms in spatiotemporal terms (of the resting state) rather than in sensorimotor, affective, or cognitive terms (as related to abnormal task-evoked or stimulus-induced activity). Among others, such spatiotemporal approach to psychopathological abnormal phenomena claims that the spatiotemporal alterations of the resting state and its internally-directed processing are manifest in abnormal experience of time and space as well as of self, other and body (Stanghellini, 2009; Stanghellini et al., 2014a, 2016).

The aim of this article is to apply this approach to psychopathological abnormalities of the body in schizophrenia. We conceive psychopathological symptoms of the body neither in cognitive nor in sensorimotor (or affective) terms, but trace them to abnormal spatial and temporal features of the resting state and its internally-directed processing. The first part of the article will briefly explain and sketch our methodological approach namely how to link the brain’s resting state to abnormal subjective experiences and psychopathological symptoms. The second and third part will shed some detailed light on the brain’s resting state activity and how its internally-directed processing are related to time in general (second part) and self/body in particular (third part). That provides the ground for conceiving psychopathological abnormalities of the body in schizophrenia in spatiotemporal terms (fourth part of this article). Finally, a fifth part shall sketch some implications of such approach. It shall be mentioned that, due to space constraints, we will not be able to discuss other approaches like the neurophenomenological approach (Thompson, 2007; Fazelpour and Thompson, 2015) that also aims to link brain and experience (in a slightly different way though than the way we aim to do; see Appendix 1 in Northoff (2014b) for discussing the neurophenomenological approach as distinguished from a neurophenomenal approach as also suggested here).

## Method—Spatiotemporal Approach to Brain and Experience

### Bottom-Up Approach: From the Brain’s Spontaneous Activity Over its Pre-phenomenal Features to the Phenomenal Features of Experience

Methodologically, spatiotemporal psychopathology includes two central features: first, the investigation of the patients’ brain and its spontaneous or resting state activity in terms of the spatiotemporal features of its internally-directed processing, and secondly, the investigation of the patients’ subjective experience of themselves and the world in predominantly spatiotemporal terms such that it can be linked directly to the former. While the first is done in neuroscience (Northoff, 2014a,b, Northoff, 2015a,b), the second requires a phenomenological approach (Stanghellini and Rossi, 2014). At first glance one may be puzzled to combine phenomenological investigation of subjective experience with neuroscientific characterization of the resting state since both cover different and seemingly mutually exclusive domains: neuronal activity, e.g., brain, and subjective experience, e.g., selfhood and personhood. In order to make direct link between the subjective experiences’ phenomenal features and the resting state’s internally-directed processing possible, we need to reveal some features that commonly underlie both, are shared, and can therefore provide the hitherto missing link. We assume that these commonly shared and underlying features are spatiotemporal features that structure and organize both the brain’s resting state and the person’s subjective experience.

The investigation of spatiotemporal features in the brain’s resting state can be done in more or less a direct way by investigating spatial variables like functional connectivity within and between regions/networks and temporal measures as frequency fluctuations and variability (Fox et al., 2005; Deco et al., 2013; Cabral et al., 2014; Northoff, 2014a,b, Northoff, 2015a,b; Smallwood and Schooler, 2015; we need to take into account several not yet fully resolved methodological issues like heart and respiration rate control and global signal regression; Duncan and Northoff, 2013; Weinberger and Radulescu, 2016). We assume that the resting state’s internally-directed processing and its spatiotemporal features predispose the various features of subjective experiences of self, body and taken in this sense, the resting state’s internally-directed processing and its spatiotemporal features are not merely neuronal but rather “pre-phenomenal” with the prefix “pre” indicating that they predispose the transformation of the brain’s merely neuronal activity into the phenomenal features of subjective experience. Without getting into detail here, we characterize “phenomenal” in terms of various features that are supposed to signify experience or consciousness; these include intentionality, self-perspectival organization, unity, temporal continuity, and qualia (among others; for details, see Van Gluick (2014) in Stanford Encyclopedia as well as Northoff (2014b) for details).

This can be referred to as “bottom up approach”: from the brain’s spontaneous activity (bottom) to experience, i.e., consciousness (top). The brain’s spontaneous activity and the spatiotemporal structure of its internally-directed processing predispose experience and can therefore be methodologically described as being pre-phenomenal. That though raises the questions: how does exactly the spatiotemporal structure characterize phenomenal experience, and which spatiotemporal structure surfaces in experience?

### Top-Down Approach: From Experience Over its Trans-Phenomenal Features to the Brain’s Spontaneous Activity

In order to address this question, we methodologically need first to investigate experience itself and reveal its underlying spatiotemporal structure. Methodologically, this requires the opposite approach: rather than moving bottom-up from the brain’s spontaneous activity to spatiotemporal structure (and from there to experience), we now need to move top-down from to its (underlying) spatiotemporal structure. Starting from experience, the top-down approach may then conjoint with the bottom-up approach in the spatiotemporal structure as shared and converging point between experience and brain.

This raises the following question: how do we investigate and reveal the spatiotemporal features of a person’s subjective experience? In a nutshell, this method, namely phenomenological investigation, entails a two-step procedure that reveals the phenomenal and trans-phenomenal features of experience.

The first—called *phenomenal* exploration—is the gathering of qualitative descriptions of a person’s lived experiences. For instance, a patient may describe his thoughts as alien (“Thoughts are intruding into my head”) and the world surrounding him as fragmented (“The world is a series of snapshots”). The result is a rich and detailed collection of the patients’ self-descriptions (Stanghellini and Rossi, 2014). In this way, we detect the critical points where the constitution of experience is vulnerable and open to derailments reflecting the “phenomenal features” of patients’ subjective experiences of specific contents, e.g., objects and events in themselves and the world. In short, phenomenal exploration as first step focuses on *contents*; experience is considered here in a content-based way.

The second step aims to shift to phenomenology proper in that it seeks the underlying or basic structures or existential dimensions of the life-worlds patients live in. Abnormal phenomena are here viewed as the outcome of a profound modification of human subjectivity within the world. Phenomenology is committed to attempting to discover the common source that ties the seemingly heterogeneous individual experiences or phenomena related to contents together thus targeting its underlying constitutive structures, namely spatiotemporal structures. These structures are not directly experienced by the person. We call these features *trans*-phenomenal, rather than merely “phenomenal”.

Historically, the prefix “trans” in “trans-phenomenal” alludes to the concept of “transcendental” as originating in philosophy. Kant used this concept to denote those conditions that are necessary for making something possible without being sufficient for its actual realization and manifestation.

The concept of “trans-phenomenal” targets those features that underlie and even more important, *constitute* subjective experience prior to and independent of the contents, e.g., events and objects. Taken in this way, the “trans-phenomenal features” concern those spatial and temporal features that structure and constitute the subject’s experience of self, body and objects and events in the world. This second step of phenomenological analysis, then, aims to recover the underlying spatiotemporal structures, the trans-phenomenal features that usually recede into the background and remain implicit yet operative in our subjective self- and world-experience.

Any phenomenon is viewed as the expression of a given form of trans-phenomenal matrix. Abnormal phenomena are the outcome of a profound modification of this matrix—that is, the transcendental (non experienced) structure that gives form to experience. For example, in schizophrenia, the person may lose its anchoring in the lived body as a Gestalt of organs and functions delimited from the external environment (Narayanan et al., 2014), or in temporal continuity (Sun et al., 2013a), or in anisotropic space, meaning space that is imbued with a point of view (Uhlhaas et al., 2006), or in intersubjective attunement and common sense (Uhlhaas and Singer, 2012), or in selfhood (Ranlund et al., 2014; for review, see Moran and Hong, 2011; Sass and Pienkos, 2013; Mancini et al., 2014).

We postulate that the trans-phenomenal features refer to spatiotemporal features since they constitute and construct the background of the contents of experience and of the life-world. Thereby the spatiotemporal features remain most often tacitly or implicitly in the background of our experience that is dominated by its contents where they nevertheless structure and organize the latter (see below for details). Our top-down approach thus proceeds from the contents of phenomenal experience to its underlying tacit or implicit trans-phenomenal background which is constitutive of experience itself. The trans-phenomenal background itself is outside of what Markovà and Berrios (2015) consider a “psychiatric object” amenable to direct psychopathological analyses. None the less, its characteristics can be deduced from careful assessement and measures conducted on the phenomenal level (for details, see Stanghellini and Ballerini, 2008).

### Convergence Between Bottom-Up and Top-Down Approaches: Correspondence or Convergence Between Pre- and Trans-Phenomenal Features

Can we link and converge the “trans-phenomenal features” of experience to the “pre-phenomenal features” of the brain’s spontaneous activity? As indicated both “pre-phenomenal” and “trans-phenomenal” features both concern spatiotemporal features: the “pre-phenomenal features” are those spatiotemporal features of the brain’s resting state that are investigated via biological methods, whereas the “trans-phenomenal” features are the same events investigated via phenomenological methods. We may assume that they are (or refer to) the same event detected and thus described in different terms (neurobiological vs. phenomenological). We suggest that whenever we find a given form of spatiotemporal pattern on the pre-phenomenal level (the brain’s spontaneous activity) this will consistently converge and match with a corresponding spatiotemporal pattern on the trans-phenomenal level. The pre- and trans-phenomenal underpin phenomenal features and thus experience, and predispose and constitute subjective experience of ourselves and the world. Due to their predisposing character, alterations in the resting state’s pre-phenomenal internally-directed processing with its spatiotemporal features may translate (in a more or less direct way) into corresponding changes in the subjective experience of space, time, body, self, others, etc.

**Figure 1:**
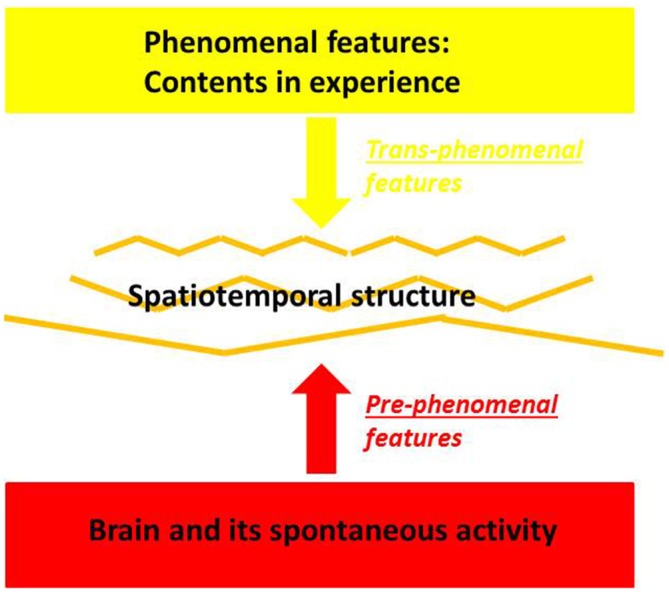
**Pre- and Trans-phenomenal features in brain and experience**.

Let us put our hypothesis into a more succinct philosophical way. The “trans-phenomenal“ and the “pre-phenomenal” are *not* two distinct levels of the living organism. They are just *methodologically* distinct. This means that to access, measure and describe these two levels we need distinct methods, e.g., bottom-up neuro-biological and top-down phenomenological approaches as described above. Note also that our claim is more empirical than ontological and taken within the empirical domain when we postulate (empirical) convergence or correspondence between the spatiotemporal features on the trans-phenomenal and the neuro-biological level. Such claim for empirical correspondence or convergence between trans-phenomenal and neuronal (or better neuro-spatiotemporal) levels does not necessarily imply their ontological identity we “pre-phenomenal” level of the brain and its spontaneous activity (with its internally-directed processing) and the “trans-phenomenal” level underlying experience by using distinct measures (see Figure 1).

Let us rephrase such spatiotemporal approach to psychopathological symptoms with regard to the concepts of the pre- and trans-phenomenal features. We suggest that these spatiotemporal abnormalities are manifest in the patients’ abnormal subjective self- and world-experience and in their respectively underlying spatiotemporal structure, the trans-phenomenal features. The latter in turn may be predisposed in the brain’s resting state and its spatiotemporal features, the pre-phenomenal features. Accordingly, we assume direct linkage from the resting state and its internally-directed processing’s pre-phenomenal spatiotemporal features over the trans-phenomenal spatiotemporal features, to abnormal spatiotemporal structuring of cognitive, affective social and sensorimotor functions as these are manifest on the phenomenal level in the various psychopathological symptoms. This shall be illustrated, specified and exemplified in the following by the example of bodily symptoms in schizophrenia.

It is important to note once more that our approach is not confined to the contents of experience. It considers abnormal contents as endpoints while focusing on their underlying construction and constitution: the contents are supposed to emerge from an abnormal underlying spatiotemporal structure, e.g., the trans-phenomenal features, which in turn are constituted in this way by abnormalities in the spontaneous activity’s pre-phenomenal features, i.e., its spatiotemporal structure. The focus on structure rather than the contents of experience distinguishes our approach from alternative hypotheseis like standard cognitive approaches (Halligan and David, 2001). There the focus on contents, and on the way these contents are associated (e.g., integration of information) by means of cognitive functions, predominates.

## Brain, Time and Experience of the Lived Body

### From Brain to Time: Operational Measures and the Spatiotemporal Structure of the Brain’s Spontaneous Activity

One should be aware that the concept of the brain’s intrinsic or resting state activity is a rather heterogeneous one and raises several methodological questions (see also Morcom and Fletcher, 2007a,b; Deco et al., 2013; Duncan and Northoff, 2013; Ganzetti and Mantini, 2013; Northoff, 2014a; Weinberger and Radulescu, 2016). Besides resting state activity, other terms like baseline, spontaneous activity or intrinsic activity are also used to describe the internally generated activity in the brain (see Deco et al., 2013; Ganzetti and Mantini, 2013; Northoff, 2014a). Even more important, the exact relationship between resting state activity and stimulus-induced or task-evoked activity remains unclear with some authors assuming mere additive interaction (Fox et al., 2005) while others presume non-linear interaction (He, 2013 Huang et al. 2015). This makes the distinction between resting state and stimulus-induced activity rather blurry so that we conceive both in relative rather than absolute terms. The terms resting state and stimulus-induced activity index extremes on a balance between internally- and externally-directed processing with a mixture of both being the “normal” case.

Resting state activity can be measured in different ways: metabolic investigations using positron emission tomography (PET) focus on measuring quantitatively the brain’s energetic metabolism indicating the resting state’s utilization and distribution of for instance glucose (Shulman et al., 2014). In contrast, fMRI as relying on the blood-oxygen-level dependent (BOLD) effects as a neuro-vascular (rather than metabolic) signal targets different resting state’s neural networks as based on statistical, i.e., correlative relationships between different regions’ voxel signifying functional connectivity (Raichle et al., 2001; Menon, 2011; Cabral et al., 2014; which may also depend on some methodological specifics such as global signal regression Saad et al., 2012; Gotts et al., 2013). Resting state activity can also concern electrophysiological or magnetic activity as measured with EEG or magnetoencephalography (MEG; Deco et al., 2013; Ganzetti and Mantini, 2013) that targets neural activity changes in different frequency ranges. Finally, one may also measure the resting state activity in psychological terms as for instance by the degree of mind wandering or spontaneous/random thoughts (Kam et al., 2013).

The different measures of resting state activity may be characterized as spatial, temporal or spatiotemporal. PET, for instance provides spatial resolution while basically showing no temporal resolution. The focus is on spatial resolution in fMRI too though functional connectivity is based on calculating time series of voxel thus introducing a temporal component. EEG/MEG show excellent temporal resolution but low spatial resolution. This makes clear-cut segregation of spatial and temporal features in resting state activity impossible entailing its integrated spatiotemporal nature.

One may want to contest that the resting state’s integrated spatiotemporal nature makes the assumption that its abnormalities are spatiotemporal almost trivially true. Resting state abnormalities are by the very nature of brain activity which is by default spatiotemporal. That is certainly true. However, the various investigations demonstrated that the resting state’s internally directed processing and its spatiotemporal features are rather dynamic and thus subject to continuous change. The resting state’s internally-directed processing continuously construct spatiotemporal features which differ from moment to moment dynamically over time. When speaking of the term “spatiotemporal” we mean such ongoing and continuous dynamic changes in internally-directed processing with the continuous construction of novel spatiotemporal features at each moment in time. Briefly, the term “spatiotemporal” is meant in a dynamic rather than static way.

Even more important, the central point in this article is not about the resting state itself and its internally-directed processing but rather about how its continuous dynamic construction of spatiotemporal features is altered in psychiatric disorders and translates into psychopathological symptoms: the abnormal spatiotemporal nature of the brain’s resting state and its internally-directed processing is supposed to directly translate into corresponding spatiotemporal abnormalities that underlie and account for psychopathological symptoms. Distinct spatiotemporal alterations in the resting state’s internally-directed processing may then be assumed to lead to distinct psychopathological symptoms.

Generally, this provides a novel perspective on psychopathological symptoms, i.e., an internally-directed resting state-based spatiotemporal perspective that complements current more externally-directed task-evoked-based sensorimotor, affective, cognitive, or social approaches (Northoff, 2015a,b). This means that psychopathological symptoms can be accounted for in terms of the spatial and temporal features of the brain’s resting state and its continuous internally-directed processing.

### From Brain Over Time to the Body: Integration of the Body’s Intero- and Proprioceptive Input within the Resting State’s Spatiotemporal Structure

**Figure 2:**
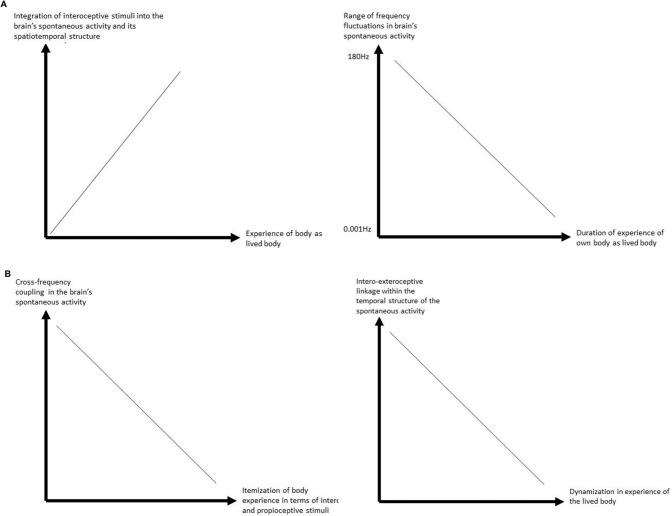
**(A)** Relationship between interoceptive stimulus processing and experience of lived body. **(B)** Relationship between interoceptive stimulus processing and itemization and dynamization of the (lived) body in experience.

The resting state is determined as the *spontaneous activity* of the brain and its internally-directed processing that *remains independent* of specific externally-directed processing and the respective external stimulus input. That though implies that there is still plenty of unspecific stimulus input from outside the brain into its resting state. This concerns exteroceptive stimuli from at least four senses which even during sleep provide continuous and unspecific input. The same applies to the body. The body is always there and provides continuous intero- and proprioceptive input into the brain and its resting state (see Figure 2).

How can we empirically support the assumption of the continuous intero- and proprioceptive input into our brain’s resting state? One would for instance expect direct correlation between the interoceptive input from the heart and resting state activity in corresponding regions. This has indeed been shown in a recent study. Chang et al. (2013) demonstrated the degree of heart rate variability directly impacted the degree of functional connectivity, i.e., its variability in the brain’s resting state activity. Especially the functional connectivity of the dorsal anterior cingulate cortex and the amygdala with subcortical regions in the brain stem, the thalamus and putamen was modulated by the degree of heart variability.

Accordingly, heart rate variability, exerts direct impact on the brain’s resting state, i.e., the variability or dynamics of its functional connectivity in those regions, i.e., subcortical and their relation to cortical ones, that show strong interoceptive input. One may even want to go further and postulate that the temporal structure of the heart’s interoceptive input may be related to and be encoded into the temporal structure of the brain’s intrinsic activity and its internally-directed processing (see for instance (Northoff, 2014a) for further details of such encoding of what can be called “vegetative statistics”). That though remains to be shown in future studies.

How do these findings and suggestions stand in relation to experience and psychopathology of the body? The interaction of the intero- and proprioceptive input from the body into the brain’s resting state activity entails that these inputs are set and integrated within the ongoing spatial and temporal dynamics, i.e., structure of the resting state. They are integrated and encoded within the resting state’s internally-directed processing and its functional connectivity (as shown above) and possibly also into its ongoing frequency fluctuations (as it remains to be demonstrated). More generally, this means that the incoming input from the body, intero- and proprioceptive, becomes integrated within a larger spatiotemporal framework of the resting state’s internally-directed processing.

How can we further support the assumption of such spatiotemporalization of the body’s intero- and proprioceptive input? This can indeed be supported on phenomenological grounds.

### From Experience to Time: “Trans-Phenomenal Temporality”

We assume that the resting-state brain activity predisposes in a pre-phenomenal way what we will call in the following transcendental temporality (TT) as specific “trans-phenomenal feature”.

The general hypothesis is that the integrity of TT is the background and condition of possibility for the integrity of the experience of time, space, body, self, and all the other dimensions of the life world. In this paragraph, we provide some introductory remarks about the concepts of lived time and TT.

We must distinguish two levels of analysis of temporality: the phenomenal and the trans-phenomenal one. On the first level we find the abnormalities of time experience. We refer to this feature of temporality with the term “phenomenal” or “lived” time. We may experience lived time as fast or slow, continuous or discontinuous, future- or past-directed, etc. The second level of temporality is *pre-thematic*, in the sense that we are not directed to it and as such it remains in the background of our phenomenal experience. It is *pre-reflectively* present. It *“functions”* implicitly and automatically. It is *passive* in the sense that it produces associative connections prior to any active engagement and is *involuntary* since it does not involve “higher” voluntary level. We refer to it with the term TT. TT underlies and constitutes any given phenomenal experience for which reason we speak of “transcendental” temporality. It is the temporal infrastructure of all experience. It has its lawful, fundamental regularity as a mode of genesis and constitution of experience.

The integrity of TT is the condition of possibility of the identity through time of an object of perception as well as of the person who perceives it. Our experience of the permanence in time of a given object whose aspects cannot exist simultaneously but only appear across time (e.g., a melody, or a tridimensional object seen from different perspectives) would be impossible if our consciousness were only aware of what is given in a punctual “now”. We can perceive an object as a unitary and identical object because our consciousness is not caught in the “now”, but the now-moment has a “width” that extends toward the recollection of past and the expectation of the future.

Time consciousness has a threefold intentional structure: primal impressions are articulated with the retention of the just-elapsed and the protention or anticipation of the just-about-to-occur. Also, the feel we have of ourselves as unitary subjects of experience remaining permanent through time is due to the integrity of TT. If we have the feel of our mental life as a streaming self-awareness this is a consequence of the continuity of inner time consciousness as the innermost structure of our acts of perception. Thanks to the unified, pre-reflexive (that is, implicit and tacit), operation of primal impression, protention and retention underlying our experience of the present our consciousness is internally related to itself and self-affecting.

Such temporal structure characterized by primal impression, protention and retention is not phenomenal since it is not experienced as such. Instead, distinguishing it from the merely phenomenal “lived time” (that is, time as it is experienced by the subject), such temporal structure, i.e., TT, is rather a “trans-phenomenal feature” that underlies and constitutes any given phenomenal experience. TT is *per se* unexperienced, thus lies on the trans-phenomenal level rather than the phenomenal as lived time. The characteristics of time experience, i.e., lived time, are simply one of the phenomenal consequences of the integrity or of the disruption of the TT. A fundamental consequence of the temporal structure provided by the integrity of TT is that we do not have partial views of ourselves and of the world, or mere isolated snapshots, or two-dimensional figures or representations, because each item of our experience is constantly integrated into a continuum which connects the present moment’s “adumbration” with retention (what we already know or have just perceived of that, or a similar, object) and protention (what we expect or imagine it to be). An “intentional arc” in consciousness bridges the retained past with the anticipated future, and thus makes possible our “milieu humain” (Merleau-Ponty, 1962, p. 158).

Conscious experience at any moment stretches from the here-and-now backwards to the past and towards the future. This function provides consciousness of the temporal horizon of the present object. No experience and no coherence of consciousness is possible without the temporal constitution of “primal presentational, retentional and protentional intentions [*urimpressionalen, retentionalen und protentionalen Intentionen*]” (Husserl, 2001, p. 233). As we have seen, there are, at least, two levels in the temporal structure of our experience: the phenomenal level, that is, the thematic articulations in the form of our active recollection and expectation, memory and imagination. And the trans-phenomenal one, that is, the implicit, pre-conceptual structuring in the form of the passive synthesis of retention and protention that we called TT. In Husserl’s terms, the constitution or construction of the structure of time is the outcome of a *passive synthesis*. Taken within our framework, “passive synthesis” may then refer to those features which we described as “trans-temporal features”. In the first place, passive synthesis occurs in the constitution of the basic temporal field in which all experience occurs. According to Husserl (2001), the basic temporal unit is not a “knife-edge” present, but a “duration-block”. It is a “constitutive flux” that that has a threefold structure. As we have seen, this temporal field is a dynamic structure that comprises the primal presentation of the now-phase articulated with the retention of the just-elapsed and the protention or anticipation of the just-about-to-occur. The structure of TT is the integration of protention-primal presentation-retention. The temporal flow of consciousness retains and protends itself and is in this way is self-unifying or, so to say, “self-synthesizing”.

We associate the term “passive synthesis” with the constitution of experience. This is in accordance with the fact that we postulate passive synthesis to constitute the trans-phenomenal features of experience. We argue that the trans-phenomenal features of experience correspond the pre-phenomenal features of the brain’s spontaneous activity. This means that the concept of passive synthesis may then also be considered within the context of the brain and thus within a neuronal rather than exclusively phenomenological context. We therefore distinguish a narrow and wide meaning of the term “passive synthesis”: the narrow meaning uses this term in an exclusively phenomenological context. The wide meaning, in contrast, extends and enlarges the concept of passive synthesis to indicate the constitution and construction of spatiotemporal features on the middle level between brain and experience, where the brain’s pre-phenomenal features converge with the experience’s trans-phenomenal features.

Synthesis’ means that something is put and linked together. We saw above that interoceptive stimuli from the body and exteroceptive stimuli from the world are linked and (knitted together in the brain’s resting state). Most importantly, this also means that the different time scales of the respective stimuli (including both internal, (or interoceptive) and external (or exteroceptive) are linked together. For instance, the 1 s scale of the interoceptive stimuli from the heart (as reflecting the delta frequency range) are integrated with the longer time scales of for instance our view of an elephant moving by (which may last around 10–20 s thus covering the infraslow range like slow four). These different time scales of intero- and exterocepetive stimuli are now linked and integrated within the brain’s resting state activity which leads to the construction or synthesis of a particular temporal structure. One may consequently want to characterize the brain’s resting state by synthesis of different time scales. Based on empirical data, we assume that such construction of different time scales by the brain’s resting state and its different frequency fluctuations predisposes the synthesis of the threefold temporal structure as described by Husserl (2001; for details, see Northoff, 2014b).

The synthesis is not subject to active construction and determination by the person himself. In other terms, the synthesis is not voluntary (and non-automatic) but involuntary (and automatic) occurring by default. This is exactly the case in the brain’s resting state and its continuous construction of time during internally-directed processing: it occurs in an automatic way by default and therefore does not underlie our active and voluntary (usually cognitive) control. Hence, the resting state’s continuous construction of time and temporal structure during its internally-directed processing can indeed by described as passive and synthetic (see chapters 13–15 and Appendix 2 in Northoff, 2014b for details as well as Northoff, 2015a). Taken in this sense, the notion of passive indicates that such synthesis occurs by default, e.g., in automatic way.

### Passive Synthesis and Body: From Passive Synthesis of Time to the Experience of Lived Body

We so far tried to demonstrate how the brain’s spontaneous activity and its internally-directed processing constructs a specific temporal structure within which the intero- and proprioceptive stimuli from the body are passively synthesized. We then went on to show the transcendental structure of experience and characterized its underlying construction processes by the concept of “passive synthesis”. This leaves now open the final step how such passive construction or synthesis of a temporal structure in both spontaneous activity and experience leads to the experience of the body as lived body. This is the focus in this section.

Since the beginning of the 20th Century, phenomenology has developed a distinction between lived body (*Leib*) and physical body (*Koerper*), or body-subject and body-object. The first is the body experienced from within, my own direct experience of my body in the first-person perspective, myself as a spatiotemporal embodied agent in the world, the second is the body thematically investigated from without, as for example by natural sciences as anatomy and physiology, a third person perspective. Phenomenology conceives of the lived body as the center of the experience of my self, and especially of the most primitive form of self-awareness, as the perspectival origin of my experiences (i.e., perceptions or emotions), actions and thoughts. The lived body has also the power of organizing experience.

Husserl (1912–1915) showed that a modification in one’s lived body implies a modification in the perception of the external world: “The shape of material things as aistheta, just as they stand in front of me in an intuitive way, depends on my configuration, on the configuration of the experiencing subject, refers to my own body”. By means of the integrity of kinaesthesia—the sense of the position and movement of voluntary muscles—my own body is the constant reference of my orientation in the perceptive field. The perceived object gives itself through the integration of a series of prospective appearances.

The lived body is not only the perspectival origin of my perceptions and the locus of their integration, it is the means by which I own the world, insomuch as it structures and organizes the chances of participating in the field of experience. The living body perceives worldly objects as parts of a situation in which it is engaged, of a project to which it is committed, so that its actions are responses to situations rather than reactions to stimuli. Last but not least, the lived body is also at the center of intersubjectivity if we understand intersubjectivity as intercorporeality, i.e., the immediate, pre-reflexive perceptual linkage between my own and the other’s body through which I recognize another being as an alter ego and make sense of her actions (Stanghellini, 2009).

How now can we link the experience of the body as lived body to the brain and its spatiotemporal structure during internally-directed processing? Based on the above sections, this leads to postulate what can be described as the “temporal hypothesis of the lived body”. We tentatively postulate that the difference between objective vs. lived body in experience is closely related to the resting state’s spatiotemporal features during internally-directed processing: the better the body’s intero- and proprioceptive input is integrated into the resting state’s ongoing temporal structure during its internally-directed processing, the higher the degree of subjective experience of the body as “lived body” as distinguished from the experience of a merely “objective body”. Though awaiting empirical support, this can be well tested experimentally in the future by combining temporal measures of the neuronal processing of interoceptive stimuli (like measuring variability in different frequency fluctuations of the brain’s spontaneous activity and how that relates to the timing of the interoceptive stimuli from for instance heart beat or respiration rate) and temporal measures for the subjective experience of the body.

Specifically, we hypothesize that the temporal dimensions in our experience of the lived body as for instance the subjectively experienced durations of the experience of the body in first-person perspective (as distinguished from the objective observation of the body in third-person perspective) may correspond to the duration of the predominant frequency fluctuations into which the body’s intero- and proprioceptive input is integrated during specifically the resting state and its internally-directed activity: the lower and stronger the brain’s frequency fluctuations that primarily integrates the body’s proprio- and interoceptive input, the longer the periods of first-person perspectival experience of the own body as lived body.

In the following, we will focus on the temporal underpinnings of our experience of the lived body. This is to neglect many other relevant dimensions like the spatial and subjective dimensions: the body as lived body is not only about time but also space. Moreover, we leave out the question: “how and why is it possible for us to experience our body as lived body at all?” Finally, we did not touch upon the subjective character of the body as our *own body* and thus in a self-related way. The discussion of these issues is beyond the scope of this article though and has been detailed elsewhere (Northoff, 2014b).

## Abnormalities in Experience of Time and Lived Body in Schizophrenia

How about psychopathological abnormalities in the experience of the lived body? Our “temporal hypothesis of the lived body” points out that alterations can occur at different levels. Either the changes can occur primarily at the level of the body which then sends abnormal intero- and proprioceptive input to the brain and its spontaneous activity; this may be the case in various medical disorders like heart disease that often go along with strong psychological symptoms like anxiety. Or, alternatively, the primary abnormality may lie in the brain itself whose spontaneous activity may construct an abnormal temporal structure which renders the subsequent integration of the body’s intero- and proprioceptive stimuli also abnormal. This, as we will demonstrate in the following, seems to be the case in schizophrenia.

For that purpose, we first pursue a top-down approach of the experiential abnormalities in schizophrenia with regard to the lived body. We first sketch abnormal experience of the lived body in schizophrenia which will be accompanied by discussing abnormal experience of time in these patients. That in turn serves to account for the abnormal experience of the lived body in terms of an abnormal underlying temporal structure reflecting what we described above as trans-temporal features. Such temporal top-down approach to the experience of the lived body will then be complemented by a bottom-up approach from the brain’s spontaneous activity and its pre-phenomenal abnormalities.

### Abnormal Experience of the Lived Body

In general, persons with schizophrenia experience throughout the course of their illness that the body loses its ambivalent status of being both an anonymous, physical object (a body as an object among other objects) and an integral part of our subjective experience (a personal body or lived body). The “vital” or “organic” part of embodiment becomes objectified: “I am provided with an anal expeller”, “arms are just prostheses”, “hands disjointed from arms”, “[I am] a bionic creature”, “a second body growing inside me”, “eyes are videocameras”, “instincts directed by electrodes”.

As the body transforms into a deanimated object, the self loses its otherwise inescapable connection to the body, it becomes a purely spiritual person, that is, a person with only mental, intellectual dimensions who considers herself as having (not being) a body, possessing it, and accordingly having complete voluntary control over this animal part of her being: “like a cybernaut in my body”, “push button to activate brain”, “supervisor of my animal body”, “all these hairs … animal body”, “[in my body] like an emperor in his pyramid”, “supernatural powers”.

There are two main properties of abnormal bodily experiences in schizophrenia: dynamization of bodily boundaries and construction, and morbid objectivization/devitalization (Stanghellini et al., 2012, 2014a,b; Madeira et al., 2016).

#### Dynamization

Patients complain about their body being violated by entities or forces coming from without their own bodily boundaries, e.g., about the intrusion or incorporation of extrapersonal things, forces, and events. This is a perplexing metamorphosis in one’s corporeal borders and *Gestalt*. Violation typically entails dynamism in the sense of experiencing something moving into oneself, not merely the static presence in oneself of something that should occupy a position external to the self. Patients also experience a dynamization of bodily construction. This is an experience of body disintegration which involves a shifting around of the usual spatial relationships between body parts, or a dynamic distortion of body *Gestalt*, i.e., of one’s body as a unitary and integrated structure. Parts of the body are felt as moving away from their usual position. Their body structure is being disintegrated and bodily parts are itemized (i.e., disaggregation of bodily parts or dissolution/loss of coherence of bodily structure).

A third aspect of bodily dynamization is the experience of externalization, that is, feeling one’s body or parts of it projected beyond one’s ego boundaries into the outer space. As is the case with violation and distortion of body construction, also externalization is not a static experience but it implies movement. Ego and corporeal boundaries, so to say, are violated from within by parts of the body that are felt as expelled into the outer space. In this type of experience as well, parts of one’s body are experienced as thing-like entities in an outer space.

#### Thingness/Mechanization

The other characterizing feature of anomalous bodily experiences is an uncanny morbid objectivization and devitalization of the body or its parts. In morbid objectivization, parts of one’s body that are usually silently and implicitly present and at work become explicitly experienced. Typically, morbid objectivization goes together with the experience of devitalization, that is, parts of one’s body are felt as devoid of life and/or substituted by some kind of mechanism. In general, the body or its parts are experienced as mere things or, thing-like entities, rather than as living flesh. Parts of oneself are spatialized—experienced as if they were dis-integrated from the living totality of one’s body. Persons with schizophrenia typically describe their condition as that of a deanimated body (“Heart no more there”, “Brain into ashes”, “Nerves as strings pulling me up”), or a disembodied mind (“I am like fog on quagmire”, “Just ethereal, no body”).

On the other hand, one may feel like a scanner or disincarnated mind which lives as a mere spectator of one’s own perceptions, actions, and thoughts. Acts of perception themselves are no more experienced from within, but from without, becoming objects of noetic awareness (“It was as if I could see my eyes watching the scene”, “I was like a receptor of stimuli”). The self breaks down into an experiencing I-subject contemplating an experienced I-object while the latter is acting or perceiving (“[My] eyes watching TV”, “[My] hand masturbating”). The phenomenality of this experience is no longer implicitly embedded in itself, that is, characterized by a pre-reflective self-awareness. In other words, the act of experiencing turns out to be an explicitly intelligible object.

The intimate, pre-reflective (that is, implicit and operative) awareness of my perceptions, actions, and thinking as *my own* is replaced by a second-order noetic (that is, explicit and conscious) awareness of something which perceives that I am perceiving, acting or thinking: “I am the spectator of my body split from the world”, “One part of the brain talks to the other”, “The world is an illusion because it is seen through a brain”. Persons with schizophrenia often describe their condition as that of a deanimated body (“Heart no more there”, “Brain into ashes”, “Nerves like strings pulling me up”), or a disembodied mind (“[I am ] like fog on quagmire”, “Just ethereal, no body”). On the face of it, such self-descriptions may seem metaphorical, but they contain a bodily “organic echo” which reveals how these persons are actually feeling and experiencing. On the one hand, one’s existence feels like that of a cyborg or a lifeless, purely mechanical body (“[I felt] like a puppet”, “No emotions, just impulses”, “[the body] a mechanical engine”, “I didn”t move it [the body] … it moved me”).

### Abnormal Experience of Lived Time

Schizophrenic persons typically describe their sense of temporal reality as: “things to a standstill”, “immobility, but not calm”, “time going back to same moment over and over”, “people like statues”, “frozen moment”, “out of time”, “marmoreal”, “unreal stillness”. Time is fragmented, there is a breakdown in time Gestalt, and an itemization of now-moments. The mere succession of conscious moments as such cannot establish the experience of continuity. Another typical phenomenon is that a revelation is on the verge to happen, the world is on the verge of ending, a new world is coming, one’s own life is on the point of undergoing a radical change. Time is “a state of suspense”, “pregnant now”, “being is hanging”, “something imminent”, “something … I didn’t know what … was going to happen … between inspiration and expiration” (Stanghellini and Rosfort, 2013).

The main feature of abnormal time experience in schizophrenia is Disarticulation—a breakdown of the synthesis of past, present and future. Disarticulation of time experience includes four subcategories.

#### Disruption of Time Flowing

Patients live time as fragmented. Past, present and future are experienced as disarticulated. The intentional unification of consciousness is disrupted. The present moment has no reference to either past or future. The external world appears as a series of snapshots. Typical sentence: “World like a series of photographs”.

#### Déjà vu/Vecu

Patients experience places, people and situations as already seen and the news as already heard. This abnormal time experience entails a disarticulation of time structure as the past is no more distinguishable from the present moment. The already-happened prevails. Typical sentence: “When I heard news I felt I had heard it before”.

#### Premonitions About Oneself

Patients feel that something is going to happen to them or that they are going to do something. This abnormal time experience entails a disarticulation of time structure as the immediate future intrudes into the present moment. The about-to-happen prevails. Typical sentence: “I felt something good was going happen to me”.

#### Premonitions About the External World

Patients feel that something is going to happen in the external world. As the previous one, this abnormal time experience entails a disarticulation of time structure as the immediate future intrudes into the present moment. The about-to-happen prevails. Typical sentence: “Something is going on, as if some drama unfolding” (Stanghellini et al., 2016).

### Disintegration of TT as Core Disorder

We assume that the disintegration of time experience on the phenomenal level reflects the disarticulation of TT on the trans-phenomenal level. The manifold of the phenomenal anomalies that we found in the life-world of persons with schizophrenia—including anomalies of phenomenal consciousness (e.g., disintegration of the appearance of external objects and itemization of external world experience), selfhood (e.g., disruption of the implicit sense of being a unified, bounded and incarnated entity), and sociality (e.g., breakdown of one’s sense of being naturally immersed in a meaningful flow of social interactions with others; Stanghellini et al., 2016)—can be traced back to a fundamental trans-phenomenal abnormality, namely the disintegration of TT. We thus pursue the second step of our top-down approach in schizophrenia, stepping down from the contents of experience, e.g., body and time, to the underlying temporal features, the trans-temporal features, that structure and organize the contents and thus the background within which the latter can be experienced.

We argue that the fragmentation of bodily experience taking place on the phenomenal level is originated by a fragmentation of TT—that is, of the pre-reflexive synthesis of impression-retention-protention. The latter takes place on the trans-phenomenal, non-experienced level and is closely related to anomalies of the resting state, which in their own turn take place on the pre-phenomenal level.

There are two features of bodily experience related to a breakdown of TT. The first is that if TT breaks down, increased heart rate, bodily tension, to impulse to flee away, blurred vision due to midriasis will appear in one’s field of consciousness as single items disconnected form each other, unrelated to one’s own self. The second one is that they will also appear as unrelated from their source in the outside world. TT is the condition of possibility for coordinating intero- and proprioceptive feelings between themselves, and with exteroceptive ones. This means that TT is needed for experiencing these phenomena as belonging to my own embodied and situated self as a spatiotemporal entity.

Bodily feelings that are no longer embedded in one’s global awareness of one’s own body (coenesthesia) are experienced as, e.g., uncanny bodily sensations (coenesthopathies), automatic movements, made impulses and actions, motor blockages, etc. Coenesthesia (*koiné aesthesis* literally means the faculty that brings together different senses) is the name for the integration of the manifold of impressions coming from one’s own body, including phenomena related intero- and exteroceptive functions, like neurovegetative functions (e.g., increase in heart rate), emotions (e.g., bodily feelings of tension), voluntary movements (e.g., fleeing), involuntary movements (e.g., midriasis), etc.

The origin of these symptoms could be searched for at the basic level where the temporal coherence of conscious awareness is constituted. A failure of the constitutive temporal synthesis may create micro-gaps of conscious experience. Feelings or sensations that are no longer embedded in the continuity of basic self-experience may appear in consciousness as “erratic blocks” and experienced as being inserted, or externalized. This is known in the clinic of schizophrenia as permeability of Ego boundaries. This coheres with the hypothesis that a breakdown of temporality may be bound up with the breakdown of pre-reflexive self-awareness.

As to the second phenomenal feature, the disruption of TT will make intero- and proprioceptive phenomena appear with no connection with events and situations in the surrounding world. For instance, disesthesic paroxisms when in stressful situation will not recognized as the manifestations of a stress reaction o an emotion elicited by the situation itself. The coupling between bodily sensations and life situation needs the integrity of TT as these are two moments in the spatiotemporal self-world relation. If the continuity of temporal experience disintegrates, overarching meaningful unification of world- (e.g., threatening situation) and self-experience (e.g., anxiety) is no longer available. The outcome is that I may feel oppressed by uncanny and incomprehensible bodily sensations evoked by interpersonal contact without me being aware that they evoked by interpersonal contacts. All this may lead to typical schizophrenic phenomena as full-blown psychotic symptoms as for instance so-called bizarre delusions.

### Abnormal Temporal Structure of Brain’s Spontaneous Activity and the Experience of the Lived Body

Can we link these phenomenal features to the brain and its spontaneous activity? Without going into detail, the empirical findings show evidence for what we describe as “temporal dysbalance” and “temporal fragmentation” in the spontaneous activity during internally-directed processing (for details, see Northoff, 2014b, 2015b). Briefly, EEG and fMRI findings indicate an abnormally shift towards slower and infraslow frequency fluctuations in schizophrenia at the expense of higher frequencies like gamma amounting to “temporal dysbalance” (Uhlhaas et al., 2006; Kikuchi et al., 2011; Moran and Hong, 2011; Spencer, 2012; Ford et al., 2012; Uhlhaas and Singer, 2012, 2013, 2015; Hanslmayr et al., 2013; Sun et al., 2013a; Narayanan et al., 2014; Ranlund et al., 2014). In addition to such temporal dysbalance between higher and lower frequencies, one can also observe decreased coupling or linkage between different frequencies, e.g., cross-frequency cocupling, between lower and higher frequencies, in schizophrenia (Allen et al., 2013; Sun et al., 2013a,b). This amount to what can be described as “temporal fragmentation” in spontaneous activity.

How now are the above described trans-temporal features of the abnormal lived body experience in schizophrenia related to the abnormal temporal structure in the brain’s spontaneous activity? We described temporal fragmentation as related to decreased cross-frequency coupling. Decreased cross-frequency coupling means that the continuous interoceptive body like the heart at every second is no longer linked to and integrated with other stimuli from the body (and also the environment) that do not fall within exactly the same time range. In the healthy brain, even stimuli that occur before or after the heartbeat can be linked and integrated due to the coupling of different frequency, e.g., those related to the heart and the ones related to other stimuli. This is no longer possible once the different frequencies are no longer coupled to each other. Both heart and other vegetative stimuli are processed in a segregated way and no longer integrated and linked to each other by the spontaneous activity’s (lacking) temporal structure during its internally-directed processing. They are consequently experienced in an isolated way.

In addition to the various vegetative or interoceptive stimuli among each other, they are also no longer properly integrated and linked to exteroceptive stimuli from the environment and proprioceptive stimuli. That in turn leads to segregation between the different stimuli, extero- and interoceptive and proprioceptive. On the experiential level this means that they and their respective objects, world and body become segregated or “itemized”, as we say above. One consequently want to hypothesize that the degree of itemization as subjectively experienced is directly proportional to the degree of (lacking) cross-frequency coupling (in exactly those frequencies that allow for integrating the various interoceptive stimuli among each other and with the extero- and proprioceptive stimuli): the low the degree of cross-frequency coupling among the relevant frequencies, the higher the degree of itemization of the body as subjectively experienced.

**Figure 3:**
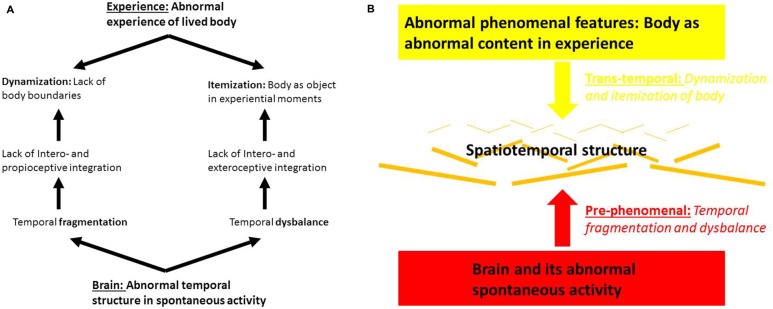
**(A)** Brain and body experience in schizophrenia. **(B)** Abnormal pre- and trans-phenomenal features in brain and body experience in schizophrenia.

Such segregated processing may then also lead to the dynamization of body boundaries as described above. If the various intero- and proprioceptive stimuli can no longer be integrated anymore, the body boundaries can no longer be clearly demarcated and distinguished from the environment. Due to lack of intero- and proprioceptive integration, the body is no longer determined as whole with clear-cut boundaries which thereby become fragile and volatile, e.g., dynamic (see Figures 3A,B).

Finally, the above described temporal dysbalance with a relative shift towards low frequencies at the expense of higher frequencies may abnormally impair the processing of stimuli in faster frequencies while stimuli in the lower range may be processed abnormally strong. This means that low- and high-frequency stimuli are processed in a dysbalanced way by the brain’s spontaneous activity: high frequency stimuli are no longer processed as strongly while low-frequency stimuli are abnormally reinforced in their neural processing by the spontaneous activity’s temporal dysbalance. In other terms low- and high-frequency stimuli from the body (and the environment) are somewhat segregated from each other. This further reinforces the split or segregation of experience into time-based units of experience, “experiential moments” that are detached from each other without any global awareness.

## Conclusion: A Spatiotemporal Approach to Brain, Experience and Psychopathology

Our article focused on the question how to link brain and experience as in the title. The purpose was not to transform a hypothesis into a law of nature with the alibi of neuroimaging techniques (Markovà and Berrios, 2015). Rather, we wanted to test a hypothesis providing converging data from psychopathological evidence (phenomenal), phenomenological contructs (trans-phenomenal) and neuroscientific measures (pre-phenomenal). Rather than laws, we here target the resting state’s capacities or predispositions (Northoff, 2013, 2015a, Northoff and Heiss, 2015) to construct a spatiotemporal structure with these capacities being abnormally altered in schizophrenia. Hence, our hypothesis is about the resting state’s capacity to construct a spatiotemporal structure for both intero- and exteroceptive stimulus processing and integration rather than to claim for imaging-based empirical generalizations, e.g., laws. The difference between laws and capacities lies mainly in the fact that the realization of capacities is strongly context-dependent with, more specifically, the brain’s spontaenous activity being dependent upon its respective environmental, e.g., socio-cultural and experiential, context (for instance, see Nakao et al., 2013; Northoff, 2014b; Duncan et al., 2015). This contrasts with the concept of laws whose instantiaton and realization remains context-independent. For the reasons of brevity, we could not go into detail about such socio-cultural and experiential context-dependence of the brain’s spontaneous activity and its spatiotemporal structure in this article. However, as it is clear, it is highly relevant for especilly psychosis and schizoprhenia where such socio-cultural and experiential context-dependence has often been observed.

We first attempted to sketched an appropriate methodological approach, a spatiotemporal approach that combines and integrates both bottom-up approaches from brain to experience and top-down approaches from experience to brain. Specifically, the bottom-up approach focuses on the brain and its spontaneous activity’s internally-directed processing and how the latter is characterized by spatiotemporal structure and their pre-phenomenal nature as predisposing certain experiential, e.g., phenomenal features. In contrast, the top-down approach starts with an analysis of subjective experience and reveals its underlying spatiotemporal features, the trans-phenomenal feature of temporality as we described it. In our tentative model, bottom-up and top-down approach converge in spatiotemporal features that allow for convergence the brain’s spontaneous activity’s pre-phenomenal features and the experience’s trans-phenomenal features.

Based on phenomenological research, the body in schizophrenia is typically experienced in an itemized way as an object external to one’s self. Based on neurobiological data, we hypothesize that such itemization of the lived body is related to decreased integration between intero-, extero- and proprioceptive stimuli by the brain’s spontaneous activity and its temporal structure during internally-directed processing. Taken all together, this suggests that we view abnormalities of bodily experience in terms of their underlying abnormal spatiotemporal features which, as we suppose, can be traced back to the spatiotemporal features of the brain’s spontaneous activity. Such “Spatiotemporal Psychopathology” (see also Northoff, 2015a,b) of the “lived body” may be developed further in the future as well as applied to other phenomenal features like experience of self, time, and space in schizophrenia and other psychiatric disorders like depression.

## Author Contributions

GN and GS contributed equally to this work.

## Conflict of Interest Statement

The authors declare that the research was conducted in the absence of any commercial or financial relationships that could be construed as a potential conflict of interest. The reviewer MB declared a past co-authorship with the author GS to the handling Editor, who ensured that the process met the standards of a fair and objective review.
